# Magnetic Dipole and Thermophoretic Particle Deposition Impact on Bioconvective Oldroyd-B Fluid Flow over a Stretching Surface with Cattaneo–Christov Heat Flux

**DOI:** 10.3390/nano12132181

**Published:** 2022-06-25

**Authors:** Seemab Bashir, Muhammad Ramzan, Hassan Ali S. Ghazwani, Kottakkaran Sooppy Nisar, C. Ahamed Saleel, Anas Abdelrahman

**Affiliations:** 1 Department of Mathematics, Air University, Islamabad 44000, Pakistan; sabbasi354736@yahoo.com; 2 Department of Computer Science, Bahria University, Islamabad 44000, Pakistan; 3 Department of Mechanical Engineering, Faculty of Engineering, Jazan University, Jazan 45124, Saudi Arabia; hghazwani@jazanu.edu.sa; 4 Department of Mathematics, College of Arts and Sciences, Prince Sattam bin Abdulaziz University, Wadi Aldawaser 11991, Saudi Arabia; drnisarks1@gmail.com; 5 Department of Mechanical Engineering, College of Engineering, King Khalid University, Asir-Abha 61421, Saudi Arabia; ahamedsaleel@gmail.com; 6 Mechanical Engineering, Faculty of Engineering & Technology, Future University in Egypt, New Cairo 11835, Egypt; anas.mohamed@fue.edu.eg

**Keywords:** magnetic dipole, gyrotactic microorganism, thermophoretic particle deposition, bioconvection, Cattaneo–Christov heat flux

## Abstract

This study emphasizes the performance of two-dimensional electrically non-conducting Oldroyd-B fluid flowing across a stretching sheet with thermophoretic particle deposition. The heat and mass transfer mechanisms are elaborated in the presence of a magnetic dipole, which acts as an external magnetic field. The fluid possesses magnetic characteristics due to the presence of ferrite particles. The gyrotactic microorganisms are considered to keep the suspended ferromagnetic particles stable. Cattaneo–Christov heat flux is cogitated instead of the conventional Fourier law. Further, to strengthen the heat transfer and mass transfer processes, thermal stratification and chemical reaction are employed. Appropriate similarity transformations are applied to convert highly nonlinear coupled partial differential equations into non-linear ordinary differential equations (ODEs). To numerically solve these ODEs, an excellent MATLAB bvp4c approach is used. The physical behavior of important parameters and their graphical representations are thoroughly examined. The tables are presented to address the thermophoretic particle velocity deposition, rate of heat flux, and motile microorganisms’ density number. The results show that the rate of heat transfer decreases as the value of the thermal relaxation time parameter surges. Furthermore, when the thermophoretic coefficient increases, the velocity of thermophoretic deposition decreases.

## 1. Introduction

In many industrial processes, working liquids have diverse rheological characteristics, whose viscoelasticity and viscosity can continually be changed and molded by exerting forces and external variables, such as stress, strain, timeframe, and temperature. These non-Newtonian fluid models are further distinguished by a nonlinear relationship between stress and deformation rates. The rate, the integral, and the differential types are the three primary classifications for these fluids. Because of the ease of mathematical modeling, many scholars have been interested in the problems of differential type fluids. In differential type models, shear stress is stated as velocity components. Nonetheless, there have been fewer attempts in the case of rate-type fluids. The Maxwell fluid model, one of the most well-known rate type fluid models, has a limited scope with only relaxation time information. However, the Oldroyd-B fluid model [1] has both relaxation and retardation time features. Ibrahim et al. [2] studied the mixed convection flow of Oldroyd-B nanofluid flow with the Cattaneo–Christov heat and mass flux model by adding mixed convection and third-order slip. Hayat et al. [3] used an exponentially stretching sheet to analyze the boundary layer flow effects of Oldroyd-B fluid. Ramzan et al. [4] discovered the effects of the magnetic dipole on ferromagnetic Oldroyd-B nanofluid flow. An Oldroyd-B nanofluid flow with heat generation and stratification are elaborated by Waqas et al. [5]. The multiple characteristics of this essential non-Newtonian fluid have attracted many researchers’ interests [6,7,8,9,10,11,12,13].

Thermophoretic particle deposition (TPD) in a liquid flow is significant in a variety of engineering procedures, such as powdered coal burner, heat exchanger, nuclear reactor protection, building ventilation systems, and air cleaners. Numerous classifications of particles act differently when subjected to a temperature gradient, resulting in the thermophoresis phenomenon. In thermophoresis, small minute particles suspended in a non-isothermal gas will attain a velocity, and this process significantly upsurges the deposition velocity of minute particles in the direction of declining temperature, but the large particles are unaffected by this process. Thermophoresis permits tiny particles to settle on a cold chilly surface. The velocity of the gas molecules in the cold region is usually less than that coming from the warm area of the particles. The particles with high velocity collide with the other particles. Then the velocity is attained by the particles due to this momentum difference, and this velocity is usually defined as the thermophoretic velocity. The thermophoretic force is exerted by a temperature gradient on suspended particles. Alam et al. [14] investigated two-dimensional steady MHD flow with thermophoresis and variable suction over a semi-infinite inclined plate in the addition of thermal radiation. Damseh et al. [15] studied thermophoresis particle deposition with the addition of MHD on a vertical surface with mixed convection. Gowda et al. [16] investigated thermophoresis particle deposition on a vertically upward downward-moving disk with a hybrid nanofluid. Kumar [17] explored the impact of the magnetic dipole on thermophoretic particle deposition, selecting Maxwell fluid over a stretching sheet. Additionally, the influence of thermophoretic particle deposition under various conditions is noticed by many authors [18,19,20,21,22,23].

Magnetohydrodynamics (MHDs) studies the dynamics of electrically conducting fluids in particular. Ferrofluids are a family of magnetizable liquids that have unique properties and a significant impact on technology. Ferrofluids are suspended magnetic particles generally measuring 10 nm distributed in a carrier liquid. Avionics, robotics, lasers, aerodynamics, computer peripherals, nuclear plants, and drawing plastic are some of the notable industrial uses of these fluids. The wide-ranging benefits of these fluids have prompted scientists and academics to mobilize their research on this specific subject. Neuringer [24] examined the magnetic dipole effects on stagnation flow point in ferrofluid at first. The presence of magnetic dipole in a ferrofluid flow was studied by Andersson and Valnes [25]. Waqas et al. [26] investigated the ferrofluid and magnetic dipole on a Carreau fluid using the Buongiorno model. In addition, references [27,28,29,30,31,32,33,34,35] address new work on ferromagnetic fluid with a magnetic dipole.

A significant element of heat transport that has been researched by many scientists is stratification. Temperature changes, concentration fluctuations, or liquids of various densities cause it in flow fields. Many engineering applications make use of the principle of stratification due to higher energy performance and efficiencies, such as industrial composition, atmospheric density stratification, and solar energy. Hayat et al. [36] investigated the effects of thermal and solutal stratification on the two-dimensional flow of an MHD Jeffrey nanofluid with the addition of mixed convection. Sandeep et al. [37] investigated dual stratification and MHD effects on a stretching sheet with the addition of a non-uniform heat source/sink. Ramzan et al. [38] studied double stratification on Williamson MHD nanofluid flow in three dimensions with Cattaneo–Christov heat flux. Rehman et al. [39] explored the mixed convection, stratification, and heat generation/absorption effects on Eyring–Powell nanofluid flow over an inclined stretching cylinder. Stratification with different flow regimes is studied by many other researchers [17,36,38,40,41,42,43,44,45,46,47].

The above-mentioned literature survey indicates that plenty of research is available considering various characteristics of nanofluid flow. However, insufficient analyses are taken into account while concentrating on electrically non-conducting Oldroyd-B nanofluid with magnetic dipole effects over a stretched sheet in addition to thermal stratification; this discussion channel becomes more focused if we add the Cattaneo–Christov heat flux model and thermophoretic particle deposition in the fluid. In this exploration, all the above-quoted aspects are added to the envisioned model. In addition, gyrotactic microorganisms of Oldroyd-B nanofluid are also employed in order to stabilize the suspended ferromagnetic particles. Furthermore, the heat transfer mechanism is better explained in the presence of a first-order chemical reaction in the current study. Using suitable similarity transformations, the governing system of a strongly nonlinear system is numerically determined. The impacts of various physical parameters on velocity, temperature, concentration, and motile gyrotactic microorganisms are calculated via graphing. The principal objective of the presented model is to answer the subsequent questions:

- What are the effects of relaxation retardation time on the velocity profile?
- How are temperature profiles affected by thermal stratification parameters?
- How is the concentration profile influenced by introducing thermophoretic particle deposition?
- How does magnetic dipole influence the skin friction coefficient?
- What is the effect of the highest swimming speed of microorganisms on the density number of motile microorganisms?

Table 1 shows the contrast comparison of the present work and the already available published work, which shows the uniqueness of the present work.

## 2. Mathematical Formulation

**Figure 1:**
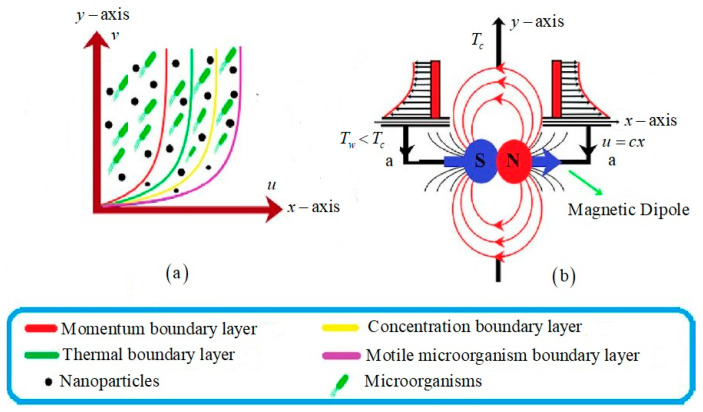
The geometry of the flow (**a**) boundary layers configuration (**b**) magnetic dipole placement.

Over a stretched sheet, a two-dimensional Oldroyd-B incompressible fluid is integrated. Due to the force applied to the sheet at *y* = 0, the sheet is stretched along the *x*-axis at the velocity *u_w_* = *cx.* A magnetic dipole is placed in the framework on the vertical axis at a distance a from the sheet. Furthermore, the magnetic dipole produces a magnetic field in the positive direction to saturate the working ferrofluid. The stretched sheet temperature *T_w_* is lower as compared to Curie temperature *T_c_*, and at this temperature, the magnetic effect vanishes. The variable temperature is *T_w_*(*x*) = *T*_0_ + *n*_1_*x*, whereas *T*_0_ is the reference temperature. Figure 1 portrays the geometrical inflow structure.

The model equations are expressed with the above-cited assumptions [10,11,12,29]:

(1) $$\frac{\partial \tilde{u}}{\partial x}+\frac{\partial \tilde{v}}{\partial y}=0,$$

(2) $$\begin{array}{l} \tilde{u}\frac{\partial \tilde{u}}{\partial x}+\tilde{v}\frac{\partial \tilde{u}}{\partial y}+{\tilde{\Lambda }}_{1}\left({\tilde{u}}^{2}\frac{\partial^{2}u}{\partial x^{2}}+{\tilde{v}}^{2}\frac{\partial^{2}\tilde{u}}{\partial y^{2}}+2\tilde{u}\tilde{v}\frac{\partial^{2}\tilde{u}}{\partial x\partial y}\right)=\frac{\mu }{\rho }\frac{\partial^{2}\tilde{u}}{\partial y^{2}}-\frac{\mu_{o}M}{\rho }\left(\frac{\partial \tilde{H}}{\partial x}\right)\\ +\upsilon {\tilde{\Lambda }}_{2}\left(\tilde{u}\frac{\partial^{3}\tilde{u}}{\partial x\partial y}-\frac{\partial \tilde{u}}{\partial x}\frac{\partial^{2}\tilde{u}}{\partial y^{2}}+\tilde{v}\frac{\partial^{3}\tilde{u}}{\partial y^{3}}-\frac{\partial \tilde{u}}{\partial y}\frac{\partial^{2}\tilde{v}}{\partial y^{2}}\right), \end{array}$$

(3) $$\begin{array}{l} \tilde{u}\frac{\partial T}{\partial x}+\tilde{v}\frac{\partial T}{\partial y}=\alpha^{*}\left(\frac{\partial^{2}T}{\partial x^{2}}+\frac{\partial^{2}T}{\partial y^{2}}\right)-\lambda_{H}\\ \left(\tilde{u}\frac{\partial \tilde{u}}{\partial x}\frac{\partial T}{\partial x}+\tilde{v}\frac{\partial \tilde{v}}{\partial y}\frac{\partial T}{\partial y}+\tilde{u}\frac{\partial \tilde{v}}{\partial x}\frac{\partial T}{\partial y}+\tilde{v}\frac{\partial \tilde{u}}{\partial y}\frac{\partial T}{\partial x}+2\tilde{u}\tilde{v}\frac{\partial^{2}T}{\partial x\partial y}+{\tilde{u}}^{2}\frac{\partial^{2}T}{\partial x^{2}}+{\tilde{v}}^{2}\frac{\partial^{2}T}{\partial y^{2}}\right)\\ -\frac{\left(T\mu_{o}\right)\partial M}{\left(\rho C_{p}\right)\partial T}\left(\tilde{u}\frac{\partial \tilde{H}}{\partial x}+\tilde{v}\frac{\partial \tilde{H}}{\partial y}\right), \end{array}$$

(4) $$\tilde{u}\frac{\partial C}{\partial x}+\tilde{v}\frac{\partial C}{\partial y}=D\left(\frac{\partial^{2}C}{\partial x^{2}}+\frac{\partial^{2}C}{\partial y^{2}}\right)-\frac{\partial }{\partial y}\left(V_{T}C\right)-k_{1}{}^{*}\left(C-C_{c}\right),$$

(5) $$\tilde{u}\frac{\partial n}{\partial x}+\tilde{v}\frac{\partial n}{\partial y}+\frac{bW_{e}}{C_{w}-C_{o}}\left(\frac{\partial }{\partial y}\left[n\frac{\partial C}{\partial y}\right]\right)=D_{m}\frac{\partial^{2}n}{\partial y^{2}},$$

with applicable boundary conditions

(6) $${\tilde{u}}_{|y=0}=cx,T_{|y=0}=T_{o}+n_{1}x=T_{w},{\tilde{v}}_{|y=0}=0,C_{|y=0}=C_{w},\;n_{|y=0}=n_{w},\;{\tilde{u}}_{|y\to \infty }=0,T_{|y\to \infty }\to T_{c}=T_{o}+n_{2}x,C_{|y\to \infty }\to C_{o},\;n_{|y\to \infty }\to n_{o},$$

where $k_{1}{}^{*}$ is the chemical reaction rate, ${\tilde{\Lambda }}_{1}$ and ${\tilde{\Lambda }}_{2}$ are relaxation and retardation times of the material, respectively, $\alpha^{*}$ is the thermal diffusivity, $\lambda_{H}$ is thermal relaxation time coefficient, *V_T_* is thermophoretic velocity, *W_e_* is highest swimming speed of microorganisms, *D_m_* is the microorganisms’ diffusion coefficient, and *n* shows the concentration of microorganisms.

## 3. Magnetic Dipole

The magnetic scalar potential for Oldroyd-B liquid flow is given by:

(7) $$\varphi =\frac{\gamma_{0}}{2\pi }\frac{x}{{\left(y+e\right)}^{2}+x^{2}}.$$

Components of the magnetic field are

(8) $${\tilde{H}}_{x}=-\frac{{\left(y+e\right)}^{2}-x^{2}}{{\left({\left(y+e\right)}^{2}+x^{2}\right)}^{2}}\frac{\gamma_{0}}{2\pi }=-\frac{\partial \varphi }{\partial x},$$

(9) $${\tilde{H}}_{y}=\frac{2x\left(y+e\right)}{{\left(x^{2}+{\left(y+e\right)}^{2}\right)}^{2}}\frac{\gamma_{0}}{2\pi }=-\frac{\partial \varphi }{\partial y}.$$

Taking

(10) $$\tilde{H}=-\nabla \varphi ,\;\tilde{H}=\sqrt{{\left(\frac{\partial \varphi }{\partial y}\right)}^{2}+{\left(\frac{\partial \varphi }{\partial x}\right)}^{2}},$$

gives

(11) $$\frac{\partial \tilde{H}}{\partial y}=\left(\frac{\gamma_{0}}{2\pi }\right)\left[-2{\left(y+e\right)}^{-3}+4x^{2}{\left(y+e\right)}^{-5}\right],$$

(12) $$\frac{\partial \tilde{H}}{\partial x}=-\left(\frac{\gamma_{0}}{2\pi }\right)\left[2x{\left(y+e\right)}^{-4}\right].$$

A linear relation between *M* and *T* is as follows:

(13) $$M=K\left(T_{c}-T\right).$$

## 4. Thermophoretic Particle Deposition

The thermophoretic particle velocity *V_T_* is given as:

(14) $$V_{T}=-\nu \kappa^{*}T_{y}\frac{1}{T}.$$

Here, κ* has the ranges of 0.2 ≤ κ* ≤ 1.2. νκ* and κ* are recognized as:

(15) $$\kappa^{*}=\frac{2C_{s}\left(\frac{\lambda_{g}}{\lambda_{p}}+C_{t}K_{n}\right)\left[1+K_{n}\left(C_{1}+C_{2}e^{-\frac{C_{3}}{K_{n}}}\right)\right]}{\left(1+3C_{m}K_{n}\right)\left(1+2C_{t}K_{n}+\frac{\lambda_{g}}{\lambda_{p}}\right)}.$$

$\lambda_{p}$, $\lambda_{g}$ are base liquid and diffused particle thermal conductivities, respectively. Additionally, C*_m_* = 1.146, C_1_ = 1.2, C*_s_* = 1.147, C_2_ = 0.41, C_3_ = 0.88, and C*_t_* = 2.20.

## 5. Similarity Transformation

Introducing dimensionless coordinates:

(16) $$\left(\xi ,\eta \right)=\left(\sqrt{\frac{c}{\nu }}x,\sqrt{\frac{c}{\nu }}y\right),\tilde{u}=cxf^{\prime }\left(\eta \right),\tilde{v}=-\sqrt{c\nu }f\left(\eta \right),$$

(17) $$\theta \left(\xi ,\eta \right)=\frac{T_{c}-T}{T_{0}-T_{w}}=\theta_{1}\left(\eta \right)+\xi^{2}\theta_{2}\left(\eta \right)=T_{c}-\left(T_{0}-T_{w}\right)\left[\theta_{1}\left(\eta \right)+\xi^{2}\theta_{2}\left(\eta \right)\right],$$

(18) $$\Omega \left(\xi ,\eta \right)=\frac{C_{c}-C}{C_{0}-C_{w}}=\Omega_{1}\left(\eta \right)+\xi^{2}\Omega_{2}\left(\eta \right)=C_{c}-\left(C_{0}-C_{w}\right)\left[\Omega_{1}\left(\eta \right)+\xi^{2}\Omega_{2}\left(\eta \right)\right],$$

(19) $$\chi \left(\xi ,\eta \right)=\frac{n_{c}-n}{n_{0}-n_{w}}=\chi_{1}\left(\eta \right)+\xi^{2}\chi_{2}\left(\eta \right)=n_{c}-\left(n_{0}-n_{w}\right)\left[\chi_{1}\left(\eta \right)+\xi^{2}\chi_{2}\left(\eta \right)\right],$$

Using the above, Equation (1) is fulfilled, and Equations (2)–(6) take the form

(20) $$f^{\prime \prime \prime }-{f^{\prime }}^{2}-\frac{2\beta \theta_{1}}{{\left(\eta +\alpha \right)}^{4}}+ff^{\prime \prime }+B_{2}\left({f^{\prime \prime }}^{2}-ff^{iv}\right)+B_{1}\left(2ff^{\prime }f^{\prime \prime \prime }-f^{2}f^{\prime \prime \prime }\right)=0,$$

(21) $$\theta_{1}^{\prime \prime }+4\theta_{2}-\mathrm{Pr}\left(f^{\prime }\theta_{1}-f\theta_{1}^{\prime }+S_{t}f^{\prime }+\lambda_{h}\left(\begin{array}{l} f^{2}\theta_{1}^{\prime \prime }+{f^{\prime }}^{2}S_{t}-ff^{\prime \prime }\theta_{1}\\ +{f^{\prime }}^{2}\theta_{1}-ff^{\prime \prime }\;S_{t}-ff^{\prime }\theta_{1}^{\prime } \end{array}\right)\right)-\frac{2\lambda \beta f\left(\theta_{1}+\varepsilon \right)}{{\left(\eta +\alpha \right)}^{3}}=0,$$

(22) $$\theta_{2}^{\prime \prime }-\mathrm{Pr}\left(3f^{\prime }\theta_{2}-f{\theta^{\prime }}_{2}-\lambda_{h}\left(\begin{array}{l} f^{2}\theta_{2}^{\prime \prime }+5{f^{\prime }}^{2}\theta_{2}\\ -3ff^{\prime }{\theta^{\prime }}_{2}-3ff^{\prime \prime }\theta_{2} \end{array}\right)\right)+\frac{2\lambda \beta f\theta_{2}}{{\left(\eta +\alpha \right)}^{3}}+\lambda \beta \left(\theta_{1}+\varepsilon \right)\left[-\frac{4f}{{\left(\eta +\alpha \right)}^{5}}+\frac{2f’}{{\left(\eta +\alpha \right)}^{4}}\right]=0,$$

(23) $$\Omega_{1}^{\prime \prime }+Sc\left(f\Omega_{1}^{\prime }-\gamma \;\Omega_{1}\right)+2\Omega_{2}+Sc\kappa^{*}N_{t}\frac{\left(N_{c}-\Omega_{1}\right)}{\left(1-N_{t}\theta_{1}\right)}\left[{\theta_{1}}^{\prime \prime }-\frac{\Omega_{1}^{\prime }\theta_{1}{}^{\prime }}{\left(N_{c}-\Omega_{1}\right)}+\frac{2N_{t}{\left(\theta_{1}\right)}^{2}}{\left(1-N_{t}\theta_{1}\right)}\right]=0,$$

(24) $$\begin{array}{l} \Omega_{2}^{\prime \prime }+Sc\left(f\Omega_{2}^{\prime }-2f^{\prime }\Omega_{2}-\gamma \;\Omega_{2}\right)-Sc\kappa^{*}N_{t}\frac{\left(N_{c}-\Omega_{2}\right)}{\left(1-N_{t}\theta_{2}\right)}\\ \left[\theta_{2}^{\prime \prime }+\frac{\Omega_{1}^{\prime }\theta_{2}^{\prime }+\Omega_{2}^{\prime }\theta_{1}^{\prime }-\Omega_{2}\theta_{1}{}^{\prime \prime }}{\left(N_{c}-\Omega_{2}\right)}-\left(N_{t}\frac{\Omega_{2}{\left(\theta_{1}\right)}^{2}+2\theta_{1}^{\prime }\theta_{2}^{\prime }}{\left(1-N_{t}\theta_{2}\right)}\right)\right]=0, \end{array}$$

(25) $$\chi_{1}{}^{\prime \prime }+Le\;f\chi_{1}^{\prime }-Pe\left(\chi_{1}^{\prime }\Omega_{1}^{\prime }-\left(\delta -\chi_{1}\right)\Omega_{1}{}^{\prime \prime }\right)=0,$$

(26) $$\chi_{2}^{\prime \prime }-Le\left(f\chi_{2}^{\prime }-2f^{\prime }\chi_{2}\right)-Pe\left(-\chi_{2}^{\prime }\Omega_{1}^{\prime }-\chi_{1}^{\prime }\Omega_{2}^{\prime }-\left(\delta -\chi_{1}\right)\Omega_{2}^{\prime \prime }+\chi_{2}\Omega_{1}^{\prime \prime }\right)=0,$$

with

(27) $$f\left(0\right)=0,\;\;\;f^{\prime }\left(0\right)=1,\;\;\;\theta_{1}\left(0\right)=1-S_{t},\;\theta_{2}\left(0\right)=0,\;\;\;\Omega_{1}\left(0\right)=1,\;\;\;\Omega_{2}\left(0\right)=0,\;\;\;\chi_{1}\left(0\right)=0,\;\;\chi_{2}\left(0\right)=0,\;f^{\prime }\left(\infty \right)=0,\;\;\;\theta_{1}\left(\infty \right)=0,\;\;\;\;\;\theta_{2}\left(\infty \right)=0,\;\;\;\;\Omega_{1}\left(\infty \right)=0,\;\;\Omega_{2}\left(\infty \right)=0,\;\chi_{1}\left(\infty \right)=0,\chi_{2}\left(\infty \right)=0,$$

and distinct dimensionless parameters are translated as under:

(28) $$\begin{array}{cc} \beta = & \mu_{o}K\frac{\gamma_{0}\left(T_{0}-T_{w}\right)\rho }{2\pi \mu^{2}},\;\;\;B_{1}={\tilde{\Lambda }}_{1}c,\;\;\;B_{2}={\tilde{\Lambda }}_{2}c,\;\;\;\alpha =\sqrt{\frac{c}{\upsilon }}e,\;\;\;\lambda =\frac{c\mu^{2}}{k\rho \left(T_{0}-T_{w}\right)},\;\;\;S_{t}=\frac{n_{2}}{n_{1}},\\ & Sc=\frac{\nu }{D},\;\;\;\lambda_{h}=c\lambda_{H},\varepsilon =\frac{T_{c}}{T_{o}-T_{w}},\;\;\;\lambda_{m}=c\lambda_{M},\;\;\;N_{c}=\frac{C_{c}}{C_{o}-C_{w}},\;\;\;N_{t}=\frac{T_{o}-T_{w}}{T_{c}},\;\gamma =\frac{k_{1}}{c},\\ & \;\mathrm{Pr}=\frac{\mu C_{p}}{k},\;Pe=\frac{bW_{e}}{D_{m}},\;\;Le=\frac{\upsilon }{D_{m}},\;\;\delta =\frac{n_{c}}{n_{o}-n_{w}}. \end{array}$$

## 6. Quantities of Practical Interest

The dimensional form of Nusselt number $N_{u}$, thermophoretic particle deposition velocity $V_{d}^{*}$, and local Stanton number $St_{r}$, and the number density of microorganisms $N_{n}$ are given by:

(29) $$N_{u}=-{\frac{xq_{h}}{k\left(T_{0}-T_{w}\right)}|}_{y=0}\;\;\;\mathrm{with}\;\;q_{h}=-{k\frac{\partial T}{\partial y}|}_{y=0},$$

(30) $$V_{d}^{*}=\frac{V_{d}}{\nu }.\;\;\;\;\;\;\;\;\;\;\mathrm{where}\;\;\;\;\;V_{d}={\frac{xq_{m}}{\left(C_{0}-C_{w}\right)}|}_{y=0}\;\;\;\mathrm{and}\;\;\;q_{m}=-{D\frac{\partial C}{\partial y}|}_{y=0},$$

(31) Vd∗=−Re12Str,    where    Str=−xqmν(C0−Cw)|y=0   and   qm=−D∂C∂y|y=0,

(32) $$N_{n}=-{\frac{xq_{n}}{D_{m}\left(n_{0}-n_{w}\right)}|}_{y=0}\;\mathrm{where}\;q_{n}=-{D_{m}\frac{\partial n}{\partial y}|}_{y=0},$$

Dimensionless *N_u_*, $V_{d}^{*}$, *St_r_*, and *N_n_* are as follows:

(33) NuRe=−(θ1′(0)+ξ2θ2′(0)),  Vd∗=−(Ω1′(0)+ξ2Ω2′(0))Sc,    ReStr=−(Ω1′(0)+ξ2Ω2′(0))Sc,NnRe=−(χ1′(0)+ξ2χ2′(0)),

where Re=cx2ν is the local Reynolds number.

## 7. Numerical Solution

For the obtained Equations (20)–(27), the MATLAB bvp4c scheme is implemented.

New variables are assumed for this purpose as:

(34) $$f(\eta )=y_{1},f^{\prime }(\eta )=y_{2},f^{\prime \prime }(\eta )=y_{3},f^{iv}(\eta )=y_{4},f^{v}(\eta )=yy_{1},\theta_{1}(\eta )=y_{5},\theta_{1}^{\prime }(\eta )=y_{6},\;\theta_{1}^{\prime \prime }(\eta )=yy_{2},\theta_{2}(\eta )=y_{7},\theta_{2}^{\prime }(\eta )=y_{8},\theta_{2}^{\prime \prime }(\eta )=yy_{3},\Omega_{1}(\eta )=y_{9},\Omega_{1}^{\prime }(\eta )=y_{10},\Omega_{1}^{\prime \prime }(\eta )=yy_{4},”\;\Omega_{2}(\eta )=y_{11},\Omega_{2}^{\prime }(\eta )=y_{12},\Omega_{2}^{\prime \prime }(\eta )=yy_{5},\chi_{1}(\eta )=y_{13},\chi_{1}^{\prime }(\eta )=y_{14},\chi_{1}^{\prime \prime }(\eta )=yy_{6},\;\chi_{2}(\eta )=y_{15},\chi_{2}^{\prime }(\eta )=y_{16},\chi_{2}^{\prime \prime }(\eta )=yy_{7}.$$

The use of the above expressions gives the following transformation to the equations:

(35) $$yy_{1}=\frac{1}{y_{1}}\left[y_{3}{}^{2}+\frac{1}{B_{2}}\left(y_{4}-\frac{2\beta }{{\left(\eta +\alpha \right)}^{4}}y_{5}-y_{2}{}^{2}+y_{1}y_{3}+B_{1}\left(2y_{1}y_{2}y_{4}-y_{1}{}^{2}y_{4}\right)\right)\right],$$

(36) $$yy_{2}=\frac{1}{\left(1-\lambda_{h}\mathrm{Pr}y_{1}{}^{2}\right)}\left[\mathrm{Pr}\left(y_{2}y_{5}+S_{t}y_{2}-y_{1}y_{6}+\lambda_{h}\left(\begin{array}{l} y_{1}{}^{2}y_{5}+S_{t}y_{2}{}^{2}\\ -y_{1}y_{2}y_{6}-y_{1}y_{3}y_{5}\\ -S_{t}y_{1}y_{3} \end{array}\right)\right)-4y_{7}+\frac{2\lambda \beta y_{1}\left(y_{5}+\varepsilon \right)}{{\left(\eta +\alpha \right)}^{3}}\right],$$

(37) $$yy_{3}=\frac{1}{\left(1+\lambda_{h}\mathrm{Pr}y_{1}{}^{2}\right)}\left[\begin{array}{l} \mathrm{Pr}\left(3y_{2}y_{7}-y_{1}y_{8}+\lambda_{h}\left(5y_{2}{}^{2}y_{7}-3y_{1}y_{2}y_{8}-3y_{1}y_{3}y_{7}\right)\right)-\frac{2\lambda \beta }{{\left(\eta +\alpha \right)}^{3}}y_{7}\\ -\lambda \beta \left(y_{5}+\varepsilon \right)\left(\frac{2y_{2}}{{\left(\eta +\alpha \right)}^{4}}-\frac{4y_{1}}{{\left(\eta +\alpha \right)}^{5}}\right) \end{array}\right],$$

(38) $$yy_{4}=Sc\left(\gamma \;y_{9}-y_{1}y_{10}\right)-2y_{11}-Sc\kappa^{*}N_{t}\frac{\left(N_{c}-y_{9}\right)}{\left(1-N_{t}y_{5}\right)}\left[yy_{2}-\frac{y_{6}y_{10}}{\left(N_{c}-y_{9}\right)}+\frac{2N_{t}y_{5}{}^{2}}{\left(1-N_{t}y_{5}\right)}\right],$$

(39) $$yy_{5}=Sc\left(2y_{2}y_{11}-\gamma \;y_{11}-y_{1}y_{12}\right)+\frac{Sc\kappa^{*}N_{t}}{\left(1-N_{t}y_{7}\right)}\left[\begin{array}{l} y_{6}y_{12}-y_{10}y_{8}-yy_{2}\;y_{11}-yy_{3}\left(N_{c}-y_{9}\right)\\ +\frac{N_{t}\left(-2y_{6}y_{8}\left(N_{c}-y_{9}\right)-y_{6}{}^{2}y_{11}\right)}{\left(1-N_{t}y_{7}\right)} \end{array}\right],$$

(40) $$yy_{6}=-Le\;y_{1}y_{14}+Pe\left(y_{14}y_{10}-\left(\delta -y_{13}\right)yy_{4}\right),$$

(41) $$yy_{7}=2Le\;y_{2}y_{15}-Le\;y_{1}y_{16}+Pe\left(-y_{10}y_{16}-y_{14}y_{12}-\left(\delta -y_{13}\right)yy_{5}+y_{15}\;yy_{4}\right).$$

With the transformed BCs:

(42) $$y_{1}(0)=0,y_{2}(0)=1,y_{2}(\infty )=0,y_{5}(0)=1-S_{t},y_{5}(\infty )=0,y_{7}(0)=0,\;y_{7}(\infty )=0,y_{9}(0)=0,y_{9}(\infty )=0,y_{11}(0)=0,y_{11}(\infty )=0,y_{13}(0)=0,\;y_{13}(\infty )=0,y_{15}(0)=0,y_{15}(\infty )=0.$$

## 8. Results and Discussion

This section depicts a graphical sketch of the involved parameters.

### 8.1. Velocity Profile

**Figure 2:**
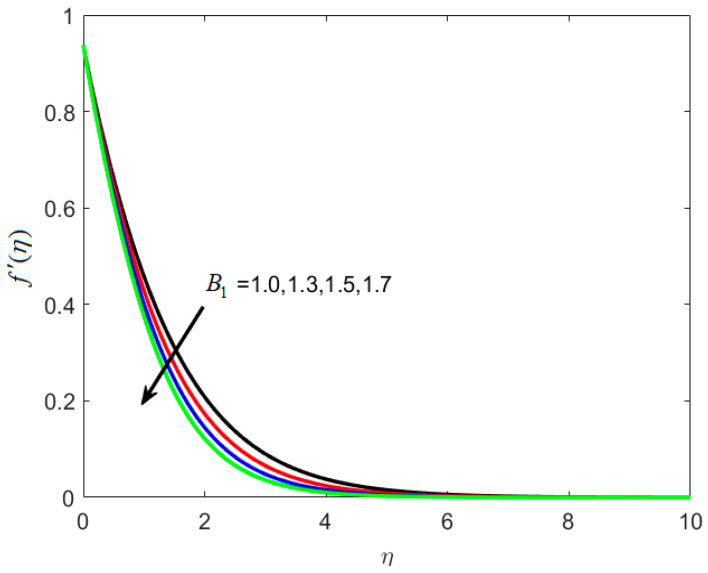
Various estimates of first material parameter $B_{1}$ by taking $B_{2}=1.2,\;S_{t}=0.3,\;\lambda_{h}=0.1,\;N_{c}=0.3,\;N_{t}=0.2,\;\gamma =0.2,\;\kappa^{*}=0.1,Le=0.2,\;\delta =0.3$.

**Figure 3:**
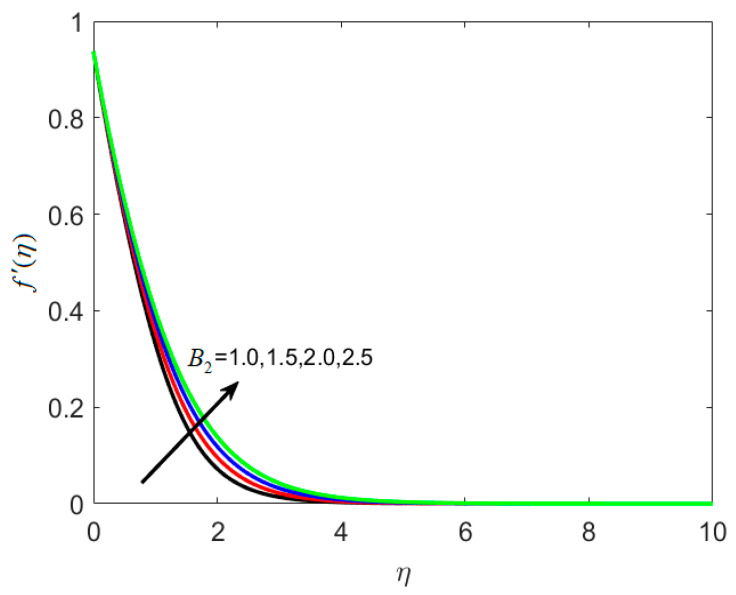
Various estimates of second material parameter $B_{2}$ by taking $B_{1}=1.2,\;S_{t}=0.3,\;\lambda_{h}=0.1,\;N_{c}=0.3,\;N_{t}=0.2,\;\gamma =0.2,\;\kappa^{*}=0.1,Le=0.2,\;\delta =0.3$.

Figure 2 shows how the material parameter $B_{1}$ affects the velocity profile. On large estimations of the relaxation time constant $B_{1}$, velocity is reduced, as seen in Figure 2. The rise in the $B_{1}$ is the reason for the reduction in velocity and causes a slower recuperation rate. The reason behind this is that for large estimates of $B_{1},$ a slower recovery process is observed, causing the thickness layer to expand at a slower pace. The effects of $B_{2}$ on $f^{\prime }(\eta )$ are seen in Figure 3. When $B_{2}$ is raised, the fluid flow is improved.

### 8.2. Temperature Profile

**Figure 4:**
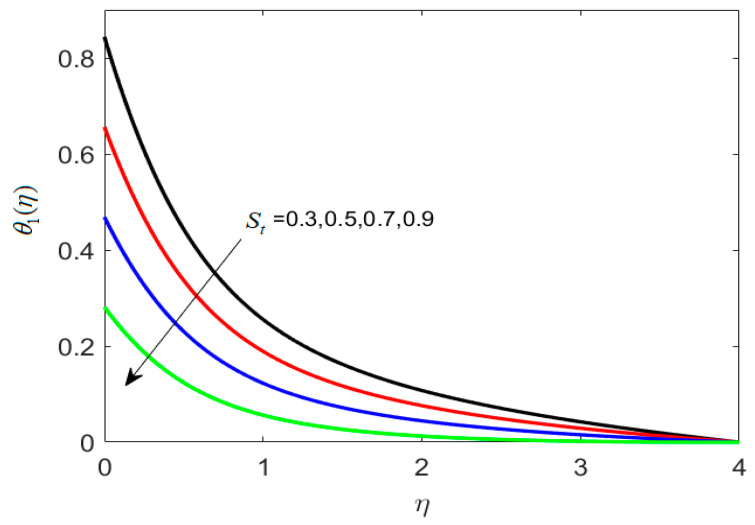
Different estimates of thermally stratified parameter $S_{t}$ by taking $B_{1}=1.2,\;B_{2}=1.3,\;\lambda_{h}=0.1,\;N_{c}=0.3,\;N_{t}=0.2,\;\gamma =0.2,\;\kappa^{*}=0.1,Le=0.2,\;\delta =0.3$.

The features of thermally stratified parameter *S_t_* against $\theta_{1}(\eta )$ are presented in Figure 4. Here temperature distribution is a decreasing function for higher (*S_t_* = 0.3, 0.5, 0.7, 0.9). In fact, $\left(T_{w}-T_{\infty }\right)$ progressively decreases for increasing *S_t_*, and hence the temperature profile $\theta_{1}(\eta )$ decreases. In addition, an increase in parameter *S_t_* causes the density of fluid layers to upsurge, resulting in dense ferrite particles to travel towards the surface, yielding increased magnetohydrodynamic interaction. This interaction causes the fluid viscosity to increase and the thermal conductivity to decrease, resulting in a reduction in heat transfer.

**Figure 5:**
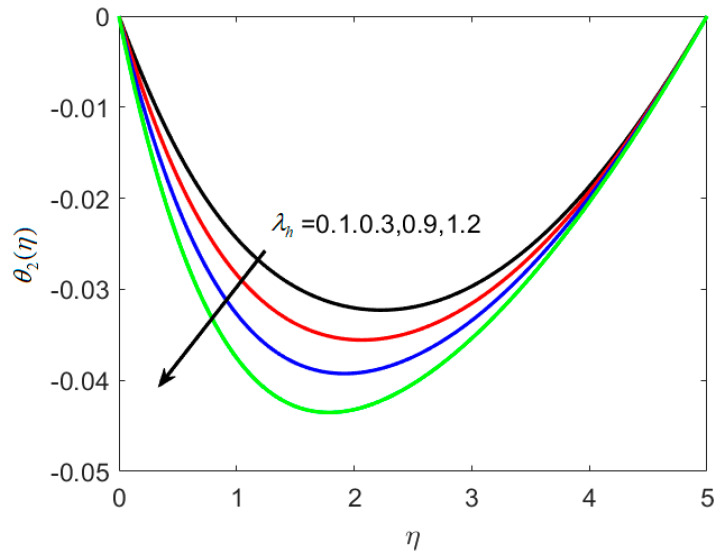
Various estimates of the thermal relaxation time parameter $\lambda_{h}$ by taking $B_{1}=1.2,\;B_{2}=1.3,\;S_{t}=0.3,\;N_{c}=0.3,\;N_{t}=0.2,\;\gamma =0.2,\;\kappa^{*}=0.1,Le=0.2,\;\delta =0.3$.

Figure 5 illustrates the consequence of the thermal relaxation time parameter $\lambda_{h}$ on $\theta_{1}(\eta )$. As seen in Figure 5, a decrease in temperature is noticed with increases in $\left(\lambda_{h}=0.3,0.5,0.9,1.2\right)$ (thermal relaxation time parameter). Because of the extended thermal relaxation period, the fluid temperature drops. This effect necessitates additional time for heat to be transported to nearby particles, which gives rise to magnetohydrodynamic interactions, and heat transfer reduces.

### 8.3. Concentration and Microorganism Profiles

**Figure 6:**
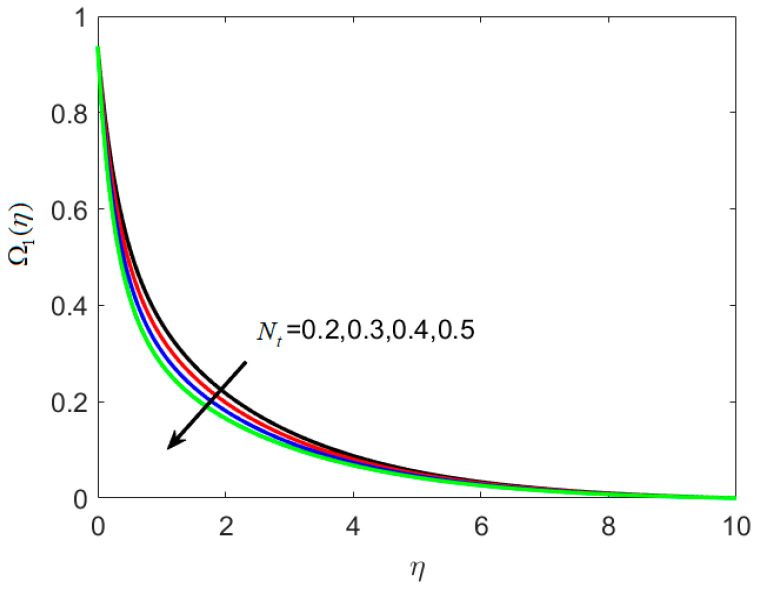
Various estimates of thermophoretic parameter $N_{t}$ by taking $B_{1}=1.3,\;B_{2}=1.2,\;S_{t}=0.3,\;\lambda_{h}=0.1,\;N_{c}=0.3,\;\gamma =0.2,\;\kappa^{*}=0.1,Le=0.2,\;\delta =0.3$.

**Figure 7:**
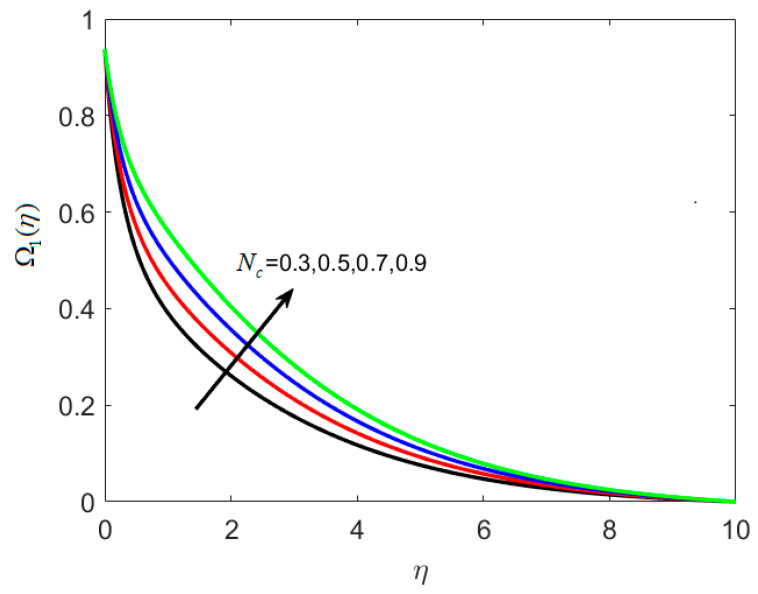
Various estimates of dimensionless concentration ratio parameter $N_{c}$ by taking $B_{1}=1.3,\;B_{2}=1.2,\;S_{t}=0.3,\;\lambda_{h}=0.1,\;N_{t}=0.3,\;\gamma =0.2,\;\kappa^{*}=0.1,Le=0.2,\;\delta =0.3$.

**Figure 8:**
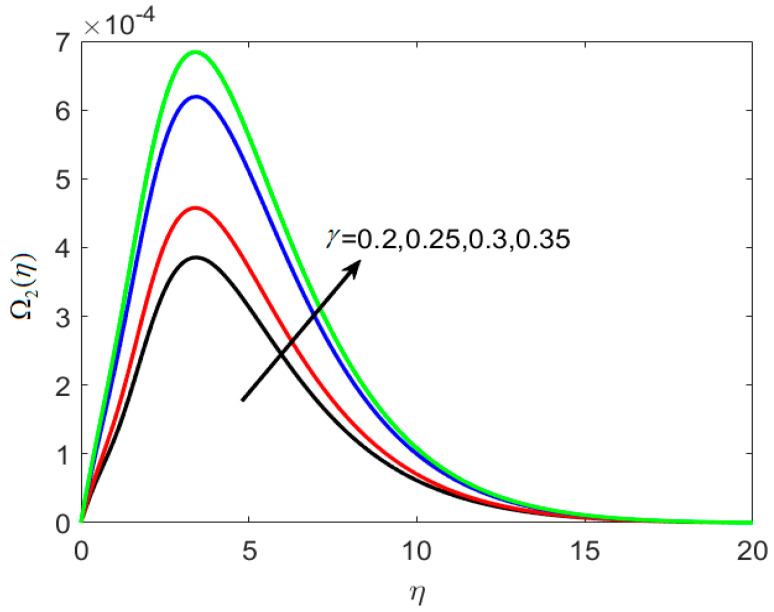
Various estimates of dimensionless reaction rate constant γ by taking $B_{1}=1.3,\;B_{2}=1.2,\;S_{t}=0.3,\;\lambda_{h}=0.1,\;N_{t}=0.3,\;N_{c}=0.3,\;\kappa^{*}=0.1,Le=0.2,\;\delta =0.3$.

On the concentration graph, Figure 6 depicts the fluctuation of thermophoretic parameter *N_t_*. It is worth noting that as *N_t_* increases, the concentration profile decreases, and the thickness of the layer decreases. In addition, when the engorged value of *N_t_* is taken, more nanoparticles are pushed away from the heated surface. The reason is that when the fluid heats up it becomes thin on the increment of thermophoresis The rise in the thermophoresis parameter has a direct influence on the flow of nanoparticles towards the cold section, resulting in a reduction in nanoparticle concentration in the fluid. Differing trends of dimensionless concentration ratio parameter *N_c_* against $\Omega_{1}(\eta )$ are seen in Figure 7. The concentration of the fluid is increased with amplified *N_c_*. The reason is that particles are engaged in the opposite path of the concentration gradient by the concentration ratio parameters, which causes the nanofluid to become more homogenous. Figure 8 is used to show the effect of dimensionless reaction rate constant γ on $\Omega_{2}(\eta )$. For large rate constant values γ, it is understood that concentration deteriorates. Large estimations of γ give a decreased concentration profile, which strengthens the decreased chemical reaction in the end.

**Figure 9:**
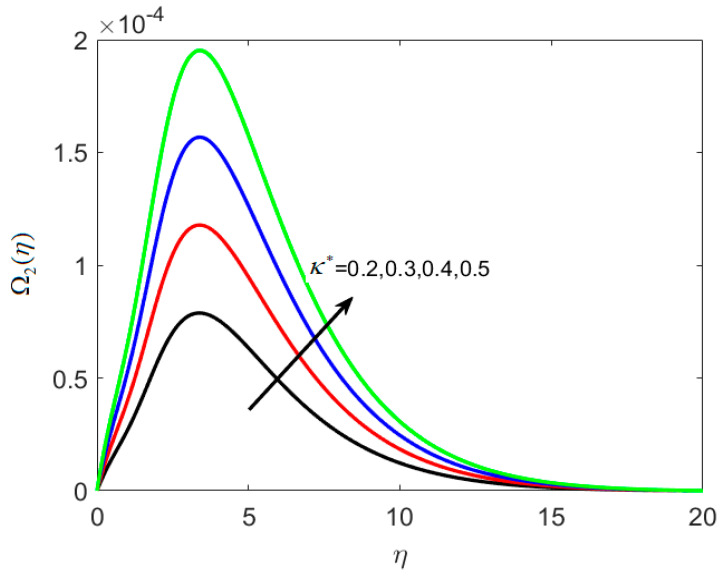
Various estimates of thermophoretic coefficient $\kappa^{*}$ by taking $B_{1}=1.3,\;B_{2}=1.2,\;S_{t}=0.3,\;\lambda_{h}=0.1,\;N_{t}=0.3,\;N_{c}=0.3,\;\gamma =0.2,Le=0.2,\;\delta =0.3$.

**Figure 10:**
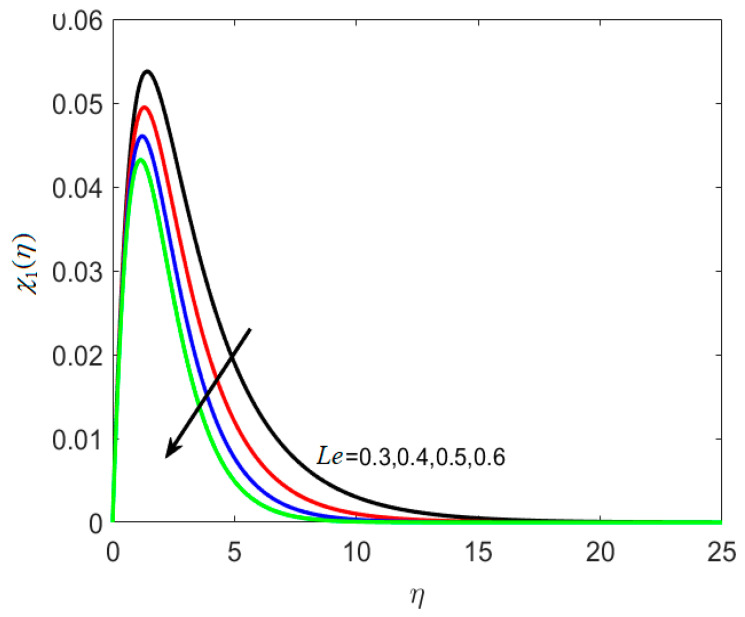
Various estimates of Lewis number Le by taking $B_{1}=1.3,\;B_{2}=1.2,\;S_{t}=0.3,\;\lambda_{h}=0.1,\;N_{t}=0.3,\;N_{c}=0.3,\;\kappa^{*}=0.1,\gamma =0.2,\;\delta =0.3$.

**Figure 11:**
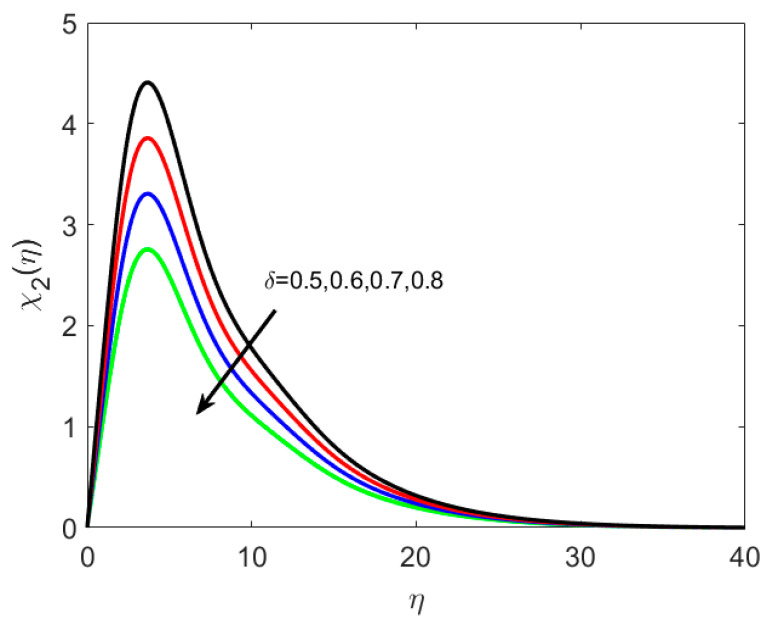
Various estimates of solutal relaxation parameter δ by taking $B_{1}=1.3,\;B_{2}=1.2,\;S_{t}=0.3,\;\lambda_{h}=0.1,\;N_{t}=0.3,\;N_{c}=0.3,\;\kappa^{*}=0.1,Le=0.2,\;\gamma =0.2$.

The concentration profile is affected by the thermophoretic coefficient, $\Omega_{2}(\eta )$, as seen in Figure 9, which is a rising function of κ* in this case. When microscopic particles are exposed to a cold surface, thermophoresis produces a suction-like effect on them. This research helps to regulate the heat gradient of a microfluidic size, which is extensively used in microdevices. Decreasing the temperature of the densest ferrite particles results in an increasing concentration boundary layer. Figure 10 shows the impact of *Le* on $\chi_{1}(\eta )$. For higher values of *Le*, the microorganisms’ diffusivity drops, and this results in the reduction of the density of liquid particles. It is illustrated in Figure 11 that boosting δ decreases $\chi_{2}(\eta )$ because the density of motile microorganisms reduces in the nanofluid flow with increasing δ. Therefore, higher δ produces a rapid reduction in the $\chi_{2}(\eta )$, because δ opposes the fluid motion.

Numerical values of *N_u_*, *St_r_*, and *N_n_* are displayed in Table 2, Table 3 and Table 4, respectively. It is evident from Table 2 that the transfer of heat rate coefficient *N_u_* decreases with increasing $\lambda_{h}$, α, and η, while its value surges with increasing β, ε, and λ. In addition, Table 3 indicates thermophoretic deposition velocity decreases with increasing *Sc* and κ*. Table 4 shows that the density number of motile microorganisms decreases with increasing Peclet number *Pe*.

Table 5 shows the comparison of $-f^{\prime \prime }(0)$ with available published work by setting $\mathrm{Pr}=1,\;\;\frac{1}{\beta^{*}}\to 0,$ and ignoring $B_{1},B_{2},\lambda ,\;\;\beta ,\;\;\lambda_{h}.$ Good agreement is observed with already published work, which increases the validity, credibility, and the accuracy of the present work.

## 9. Concluding Remarks

In this investigation, we explored the impact of magnetic dipole and thermophoretic particle deposition on Oldroyd-B fluid flow over a stretching sheet. In the proposed model, to analyze the heating mechanism, the Cattaneo–Christov heat flux model is added to an electrically non-conducting, thermally stratified ferromagnetic nanofluid. Magnetic dipole effects are also taken into account. Additionally, the concentration field is inspected under consideration of thermophoretic particle deposition and chemical reaction. Gyrotactic microorganisms of Oldroyd-B nanofluid are employed in order to stabilize the suspended ferromagnetic particles. The following are the problem’s most notable outcomes:

- The opposite behavior of velocity function $f^{\prime }(\eta )$ is observed with increasing relaxation retardation time constants *B*_1_ and *B*_2_.
- Thermal stratification parameter *S_t_* minimizes temperature profiles.
- $\Omega_{1}$ and $\Omega_{2}$ decrease with increasing *N_t_*.
- Thermal relaxation parameter $\lambda_{h}$ decreases the temperature profiles.
- *N_u_* increases with increasing β.
- Large estimations of γ decrease the concentration profile.
- Thermophoretic deposition velocity decreases with increasing *Sc* and κ*.
- The density number of motile microorganisms decreases with increasing *Pe* and *Le*.

## Figures and Tables

**Table 1:** A comparison of present work with closely comparable published research efforts.

Authors	Oldroyd-B	Magnetic Dipole	Thermophoretic Particle Deposition	Cattaneo–Christov Heat Flux	Thermal Stratification	Gyrotactic Microorganisms	Chemical Reaction
[10]	Yes	No	No	Yes	Yes	No	Yes
[17]	No	Yes	Yes	No	No	No	Yes
[29]	No	Yes	Yes	Yes	Yes	No	No
Present	Yes	Yes	Yes	Yes	Yes	Yes	Yes

**Table 2:** Estimation of Nusselt number (NuxRex12 ) for varying parameters $\lambda_{h}$, β, ε, Pr, λ, $S_{t}$, α, η.

$\lambda_{h}$	β	ε	Pr	λ	$S_{t}$	α	η	$-(\theta_{1}^{\prime }(0)+\xi^{2}\theta_{2}^{\prime }(0))$
0.5	1.1	0.1	1.2	0.1	0.1	0.3	1.1	1.1007748
0.6								1.0969643
0.7								1.0838647
	1.2							1.1008176
	1.3							1.1008605
		0.2						1.1016806
		0.3						1.1025864
			1.3					1.1460540
			1.4					1.1885233
				0.2				1.1021934
				0.3				1.1036116
					0.2			1.1906204
					0.3			1.2804782
						0.4		1.1006387
						0.5		1.1005585
							1.2	1.1006819
							1.3	1.1006387
					1.1			0.20969592
					1.2			0.2145712

**Table 3:** Numerical estimation of local Stanton number (Str Rex12) for different parameters γ, $N_{c}$, $N_{t}$, Sc, $k^{*}$.

γ	$N_{c}$	$N_{t}$	Sc	$\kappa^{*}$	$-\frac{\left({\Omega^{\prime }}_{1}(0)+\xi^{2}{\Omega^{\prime }}_{2}(0)\right)}{Sc}$
0.7	1.3	0.1	0.4	1.5	1.4893254
0.8					1.5668322
0.9					1.6394216
	1.4				1.4763646
	1.5				1.4634038
		0.2			1.4392334
		0.3			1.3787588
			0.5		1.3458035
			0.6		1.245990
				1.6	1.4864837
				1.7	1.4836393

**Table 4:** Numerical estimation of the number density of microorganisms (Nn Rex−12 ) for different parameters Le , δ , Pe.

Le	δ	Pe	$-\left(\chi_{1}^{\prime }\left(0\right)+\xi^{2}\chi_{2}^{\prime }\left(0\right)\right)$
0.2	0.5	1.1	−0.28919802
0.3			−0.28562933
0.4			−0.2809328
	0.6		−0.34703762
	0.7		−0.40487721
		1.2	−0.31631026
		1.3	−0.3435266

**Table 5:** Comparison of $-f^{\prime \prime }(0)$ with available published work by suppressing the additional parameters. Selecting $\mathrm{Pr}=1,\;\;\frac{1}{\beta^{*}}\to 0,$ and considering $B_{1}=B_{2}=\lambda ,=\;\beta ,=\;\lambda_{h}=0$.

Published Articles	$-f^{\prime \prime }(0)$
Chen et al. [29]	0.6012011
Kumar et al. [17]	0.6069352
Pal et al. [46]	0.615066
Zeeshan et al. [48]	0.6058427
Present	0.6012541

## Data Availability

The datasets used and/or analyzed during the current study are available from the corresponding author on reasonable request.

## Nomenclature

c	Constant	$\tilde{u},\tilde{v}$	Velocity components m/s
$V_{T}$	Thermophoretic velocity	$V_{d}$	Thermophoretic deposition velocity m/s
x,y	Coordinates axis m	C	Concentration $m^{-3}mol$
$N_{t}$	Thermophoretic parameter	$V_{d}^{*}$	Non-dimensional thermophoretic deposition velocity
$N_{c}$	Dimensionless concentration ratio	T	Temperature K
b	Chemotaxis constant	**Greek symbols**	
$T_{c}$	Curie temperature K	λ	Viscous dissipation parameter
$W_{e}$	Highest swimming speed of microorganisms	$\tau_{w},q_{h},q_{m}$	Shear stress, surface heat flux, surface mass flux
k	Thermal conductivity $W\;m^{-1}K^{-1}$	$\lambda_{H}$	Thermal relaxation time coefficient
M	Magnetization $m^{-2}Wb$	$\mu_{o}$	Free space permeability $A^{-2}N$
Sc	Schmidt number	${\tilde{\Lambda }}_{1},{\tilde{\Lambda }}_{2}$	Relaxation, retardation times of material parameters
f(η)	Dimensionless velocity	K	Gyromagnetic coefficient
$S_{t}$	Thermal stratification parameter	$\gamma_{0}$	Strength of magnetic field cm
$L_{e}$	Traditional Lewis number	γ	Dimensionless reaction rate constant
e	Distance cm	δ	Solutal relaxation parameter
		μ	Dynamic viscosity $m^{2}s^{-1}$
$\tilde{H}$	Magnetic field	$\theta_{1}\left(\eta \right),\theta_{2}\left(\eta \right)$	Dimensionless temperature
$T_{w}$	Wall temperature K	β	Ferromagnetic interaction parameter
$B_{1}$, $B_{2}$	Deborah numbers or dimensionless material parameters	$n_{w}$	Diffusive concentration of microorganisms at the wall
$P_{e}$	Bioconvection Peclet number	η, ξ	Similarity variables
		ν	Kinematic viscosity $m^{2}s^{-1}$
$k_{1}{}^{*}$	Chemical reaction rate	α	Dimensionless distance
Pr	Prandtl number	$\Omega_{1}\left(\eta \right),\Omega_{2}\left(\eta \right)$	Dimensionless concentration
Rex	Local Reynolds number	φ	Scalar potential
$\kappa^{*}$	Thermophoretic coefficient	ε	Dimensionless curie temperature
$St_{r}$	Local Stranton number	ρ	Density $kgm^{-3}$
D	Diffusion coefficient $m^{2}s^{-1}$	$\beta^{*}$	Fluid parameter
$N_{u}$	Local Nusselt number	φ	Scalar potential
$N_{n}$	Density of motile microorganisms	$\lambda_{h}$	Thermal relaxation parameter
$K_{n}$	Knudsen number	$\chi_{1}\left(\eta \right),\chi_{2}\left(\eta \right)$	Dimensionless diffusive concentration of microorganisms
$n_{1},n_{2}$	Constants	$\chi_{1}\left(\eta \right),\chi_{2}\left(\eta \right)$	Dimensionless diffusive concentration of microorganisms
$T_{0}$	Reference temperature *K*	$\alpha^{*}$	Thermal diffusivity $m^{2}s^{-1}$
$C_{p}$	Specific heat capacity J/(K kg)		
$C_{f}$	Skin friction coefficient		
$C_{0}$	Reference concentration $m^{-3}mol$		
